# The effect of Virtual Reality Cycling with Music on simple obesity in college students: evidence from a randomized controlled trial in China

**DOI:** 10.3389/fpubh.2024.1466142

**Published:** 2024-11-21

**Authors:** Meng Zhao, Ying Lei, Ziran Wei, Ming You

**Affiliations:** ^1^Department of Physical Education, Nanjing Agricultural University, Nanjing, China; ^2^Exercise and Health Promotion Research Center, Nanjing Agricultural University, Nanjing, China; ^3^College of Sports Training, Nanjing Sport Institute, Nanjing, China

**Keywords:** virtual reality cycling, music intervention, shaping, simple obesity, youth sport

## Abstract

**Background:**

Simple obesity has become an increasingly prevalent health concern among college students. Existing research indicates that traditional exercise methods may not sufficiently engage this population, particularly those facing barriers to regular physical activity. Thus, this study investigated how combining VR cycling with music influenced fat reduction in this population.

**Method:**

This study recruited 78 Chinese college students with simple obesity (BMI ≥ 28 kg/m^2^) and randomly assigned them to either an experimental group (VR cycling combined with music, *n* = 41) or a control group (traditional cycling, *n* = 37). Both groups engaged in 12 weeks of cycling training, three times a week, for 45 min per session. Key physiological indicators, such as weight, BMI, waist circumference, and hip circumference, were measured at baseline, mid-intervention, and post-intervention.

**Results:**

The VR cycling with music group exhibited significant improvements in key physiological metrics compared to the control group. Notable changes included a 4.1% reduction in weight, a 2.8% decrease in BMI, and a 2.8% reduction in waist circumference. Hip circumference also decreased by 2.5%, while the waist-to-hip ratio dropped by 0.016 units. Furthermore, the intervention led to a 7.2% increase in vital capacity, with all outcomes showing statistical significance (*p*<0.01). The experimental group also reported higher levels of enjoyment and engagement throughout the intervention period.

**Conclusion:**

Participants in the experimental group experienced significant reductions in weight, BMI, and both waist and hip circumferences, as well as a notable improvement in vital capacity. The study highlights that combining virtual reality cycling with music resulted in more substantial weight loss and fat reduction in obese college students, compared to traditional cycling methods.

## 1 Introduction

Obesity has emerged as a critical global health epidemic, ranking as the fifth leading cause of mortality worldwide (1). The World Health Organization (WHO) reports that by 2022, over 2.5 billion adults, representing approximately 43% of the global adult population, were classified as overweight, with more than 890 million considered obese. This marked increase from 1990, when 25% of adults were overweight, underscores the accelerating pace of this epidemic (2). Obesity is typically diagnosed using body mass index (BMI), calculated as weight (kg) divided by height squared (m^2^), which serves as a surrogate marker for body fat. A BMI of 25 or more defines overweight, and a BMI of 30 or more defines obesity (2). The health risks associated with overweight and obesity have been extensively documented and understood. These conditions are significant contributors to non-communicable diseases (NCDs), including type 2 diabetes mellitus, hypertension, cardiovascular diseases, certain types of cancer, and musculoskeletal disorders (3, 4).

Obesity is often classified into two categories: simple obesity and secondary obesity, with simple obesity accounting for around 95% of cases. Simple obesity primarily results from an imbalance between caloric intake and expenditure, leading to excess fat accumulation (5). College students are particularly susceptible to simple obesity, as this stage of life often coincides with significant lifestyle changes. These changes include decreased physical activity, shorter sleep duration, increased stress levels, and the adoption of unhealthy dietary habits, such as increased consumption of soft drinks, sweets, fried and processed foods, and irregular meal patterns. These factors collectively contribute to an increase in body fat. Furthermore, obesity trends established during collegiate period frequently lasts into adulthood, resulting in long-term health issues (6–9). A recent study found that 23.5% of male and 11.9% of female college students in China were classified as overweight or obese (10).

Given the growing number of college students in China, which reached 47.63 million in 2023 (11), addressing obesity in this population is of critical public health importance. Promoting physical activity during the university years is a key strategy in preventing and managing obesity, as exercise not only helps regulate body weight but also improves appetite control and energy expenditure (12–14). However, many college students face barriers to regular exercise, including perceived inadequacies in campus recreation facilities, time constraints, lack of motivation, and the need for social support (15, 16). Moreover, sustaining interest in physical activity is often difficult, as repetitive routines can lead to disengagement and eventual cessation of exercise (17). Therefore, innovative strategies are essential to motivate college students to consistently engage in physical activity.

Virtual reality (VR)-based exercise presents a promising solution to address the challenges of promoting regular physical activity among college students by offering an immersive and engaging experience (17, 18). Among various VR applications, VR cycling stands out, combining traditional indoor cycling with VR technology. This approach allows users to ‘ride’ through virtual landscapes, transforming an otherwise monotonous activity into a dynamic and stimulating experience (19, 20). The primary advantage of VR lies in its ability to create an immersive, computer-generated environment that users perceive as real. This sense of “being there” allows users to experience the virtual world as though they are physically there, enhancing their engagement and overall experience (21, 22). Furthermore, VR improves levels of participation by enhancing cognitive focus, concentration, and providing an enjoyable, stimulating experience (18). Compared to real-world exercise, VR-based physical activity significantly increases enjoyment and self-efficacy while reducing fatigue (23). The inherently enjoyable nature of VR fosters deeper immersion, which in turn can enhance both motivation and learning outcomes (24–26). For individuals with obesity, VR technology alleviates the discomfort often associated with high-intensity physical activity, leading to improved performance and the ability to sustain exercise for longer durations (27, 28).

From the perspective of hedonic psychology, the impact heuristic suggests that individuals are more inclined to engage in activities that evoke positive emotions and avoid those that generate discomfort (29, 30). Accordingly, individuals who experience heightened positive affect and diminished negative affect during physical activity are more likely to adhere to their exercise routines (31). Music, as an effective tool for enhancing emotional experiences, plays a crucial role in this context. It not only amplifies enjoyment but also increases the likelihood of exercise persistence, extends activity duration, and induces a more positive emotional state (32–34). Over the past two decades, extensive research has consistently shown that music amplifies emotional responses across various forms and intensities of physical activity (32–34). Music influences exercise through two primary mechanisms: rhythmic entrainment and attention dissociation (32–34). Rhythmic entrainment occurs when the music’s tempo aligns with the individual’s physical movements, optimizing motor coordination and energy efficiency. This synchronization not only enhances performance but also deepens the emotional connection to the activity by creating a more cohesive and enjoyable experience (32–34). Additionally, music promotes attention dissociation, drawing focus away from internal sensations of fatigue or discomfort and toward external auditory stimuli, reducing perceived effort. As a result, individuals can exercise for longer periods with greater endurance (32–35).

Although previous research has demonstrated the benefits of VR-based exercise in improving engagement, enjoyment, and physical performance, and the role of music in improving emotional states and extending exercise durations through mechanisms like attention dissociation and rhythmic entrainment, the combined effects of VR cycling with music on the physical responses of college students with obesity remain underexplored. In this study, we developed a new exercise intervention that integrates VR cycling with music and compared its effects to conventional indoor cycling. The combination of music and immersive VR environments is expected to further enhance user engagement and enjoyment, promoting long-term adherence to physical activity, which is particularly important for college students with obesity, where sustained exercise is crucial for effective weight management. Investigating how this combined approach influences exercise adherence, and long-term weight management in this population is critical for advancing obesity interventions and optimizing VR-based exercise programs.

## 2 Materials and methods

### 2.1 Participants

This study ultimately included 78 college students diagnosed with simple obesity from a university in the eastern region of China, with participants aged between 18 and 24 years. The participants were randomly assigned to either an experimental group (41 students: 25 males and 16 females) or a control group (37 students: 20 males and 17 females) using simple randomization. Randomization was conducted using Excel 2010 to generate random numbers, ensuring complete randomness in group allocation. Sealed envelopes were used to maintain concealed group allocation.

The inclusion criteria required participants to have no self-reported physical or psychological disabilities, and to have maintained stable weight fluctuations within 5% over the past 6 months (36). Additionally, participants were required to meet specific diagnostic criteria for simple obesity, which was confirmed by ruling out secondary obesity through medical history, physical examination, and laboratory tests. Specifically, the criteria included a measured weight exceeding 20% of the standard weight, a body fat percentage greater than 20% for males and greater than 30% for females, and a BMI greater than 28 kg/m^2^ (37).

The sample size for this study was determined through a power analysis conducted using G*Power version 3.1.9.7 software. Effect sizes were calculated using Cohen’s d, where an effect size of ≥0.20 is considered small, ≥0.50 is considered medium, and ≥0.80 is considered large. Building on previous research (38) and aiming to enhance the detection of smaller effects, this study chose an effect size of 0.3, considering the 12-week duration of the intervention. The calculation was based on repeated-measures ANOVA: the within-between interaction type I error rate was set at 5% (*α* = 0.05), the effect size of the main outcome was set at 0.3 and the type II error rate gave 95% power for the two groups and two repeated sets of measurements; correlation among the repeated measures was assumed at 0.5 and the non-sphericity correction *ε* was 1.0. Based on these assumptions, it was determined that a minimum of 40 participants per group was required. To ensure robustness and account for possible dropouts, we recruited 82 participants, including four dropouts from the control group. All participants provided informed consent before participating in the experiment.

In this study, the control group received standard recommendations for regular cycling exercise, while the experimental group participated in a 12-week virtual reality cycling program. This program was conducted at specified times and locations, with the flexibility to cycle in the gym or other approved venues under special circumstances. Importantly, no participants reported motion sickness or visual fatigue throughout the study. This study implemented a randomized control trial study design, approved by the Human Research Ethics Committees of Nanjing Sport Institute (Approval Number: RT-2022-11), and personal data confidentiality was maintained in accordance with the General Data Protection Regulation. The participants were verbally briefed on the study procedures, provided with comprehensive written information, and asked to sign a consent form. Additionally, they were encouraged to ask questions and received complete and detailed responses to ensure their full understanding.

### 2.2 Experimental design

#### 2.2.1 Requirements for VR cycling

Before VR cycling, the resistance of the dynamic bicycle should be adjusted to zero, that is, a state of no resistance. The seat height should be aligned with the cyclist’s hip joint, ensuring the knee joint remains slightly bent to reduce knee stress during cycling. For male cyclists, a slight forward tilt of the seat is recommended to enhance comfort and optimize riding posture. The handlebars should be adjusted to the same height or slightly higher than the seat to maintain an ergonomic riding position. After the cyclist is seated, the pedal straps should be secured to a comfortable tightness, and any loose shoelaces should be tucked inside the shoes to prevent entanglement. Cyclists are advised to wear gloves and keep a water bottle within easy reach.

During VR cycling, the resistance level should be modified according to the road conditions simulated in the VR environment. The feet should remain parallel to the ground, and the knee joints should maintain alignment with the feet throughout the cycling motion. A straight posture for the back and waist should be maintained, with the elbows kept slightly bent and positioned inward to reduce upper body tension. Cyclists should focus on keeping their gaze forward, avoiding unnecessary head movements. Movement should follow the rhythm of the music, and hydration should be maintained by taking small sips of water at the end of each VR segment (39, 40).

#### 2.2.2 VR cycling application

**Figure 1:**
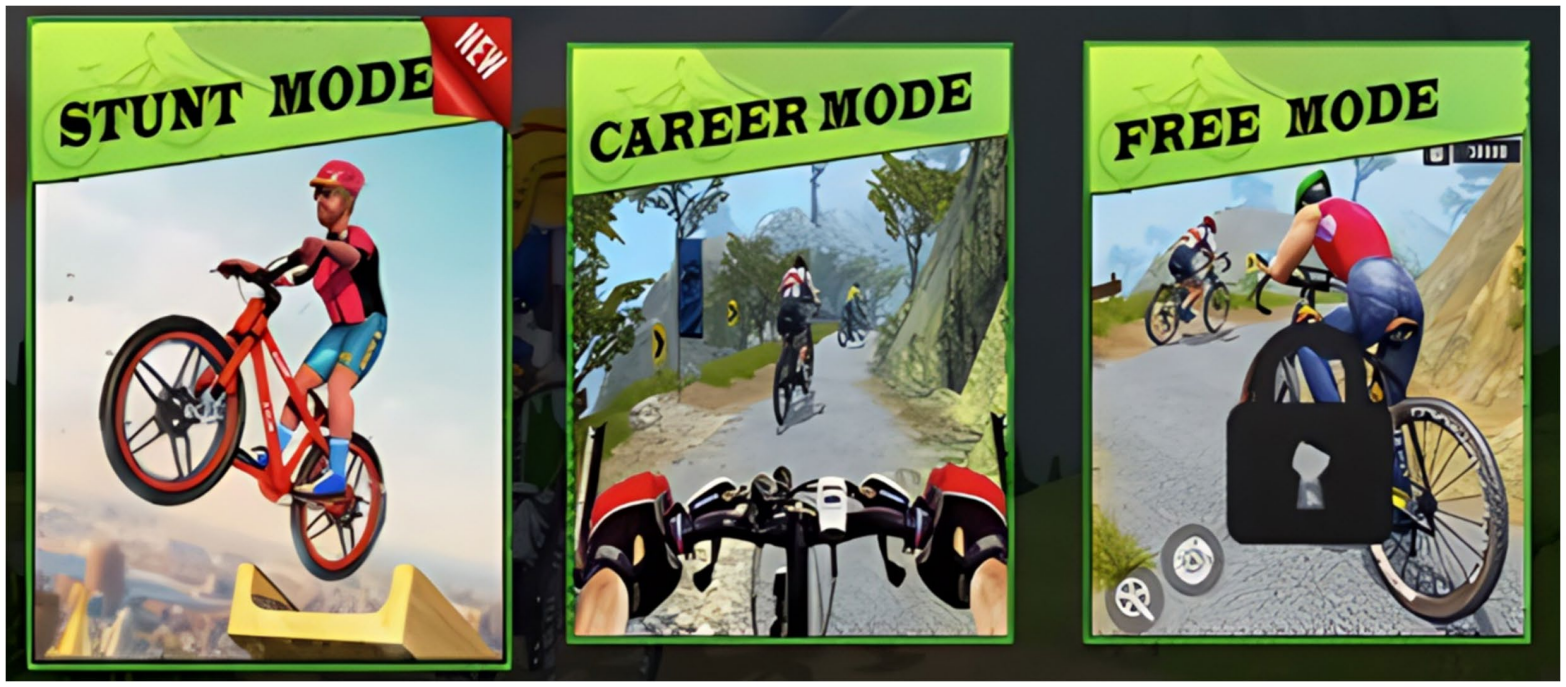
Virtual scene mode in VR cycling.

**Figure 2:**
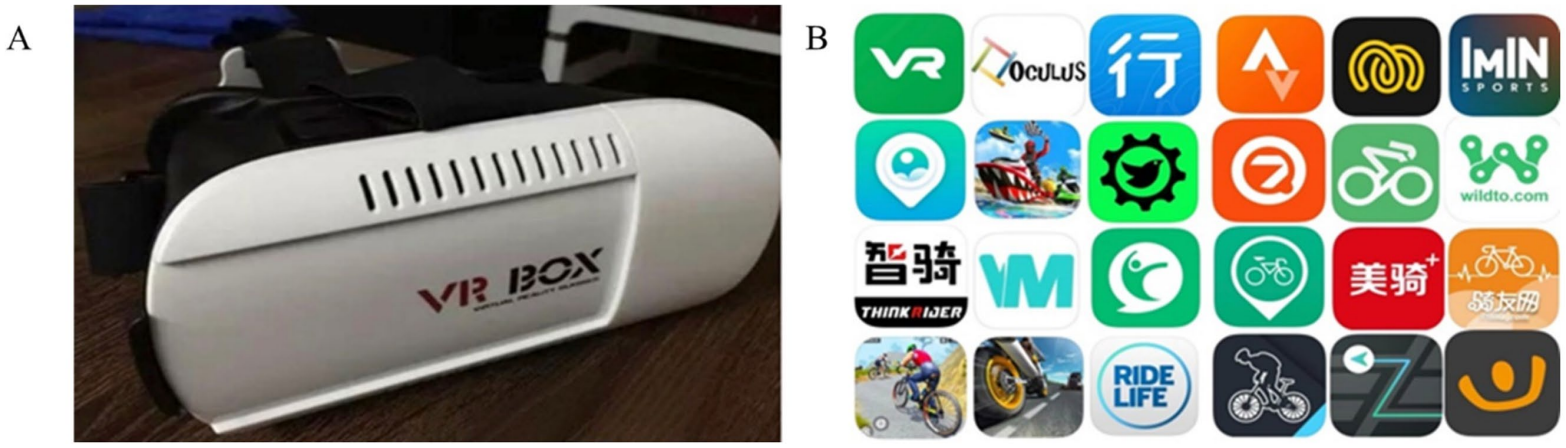
Experimental VR glasses (A) and VR cycling-related app (B).

In this study, VR goggles were used in conjunction with specialized applications to simulate immersive cycling environments. These applications provided a variety of cycling scenarios, such as urban streets, mountainous terrain, and forest trails, offering a dynamic and engaging experience for the participants (see Figure 1 and Supplementary Figure S1). Each session was tailored by coaches to align with the weekly training goals, and participants cycled through different road scenes selected based on the content of the session. To enhance the immersive experience, dynamic music of various styles was integrated with the VR environment, corresponding to the intensity and mood of each cycling scenario (41). Each cycling session incorporated a standardized structure that included essential exercises such as warm-up, flat road standing cycling, high resistance climbing, seated sprint, intermittent cycling, stretching, and cooling down (42). The VR goggles and the applications used in this study are shown in Figure 2. These VR tools provided not only visual immersion but also physical engagement, as participants adjusted their resistance levels based on the simulated terrain (43).

#### 2.2.3 Music selection

Each 45-min training session was accompanied by carefully selected music (44). The music used was pre-edited into specific segments, with the rhythm aligned to support various exercise intensities and VR scenarios. During the warm-up phase, lasting 6–7 min, slow-tempo music with a relaxing rhythm was chosen to promote a gradual increase in heart rate and a calm mental state (45), preparing participants for the upcoming physical effort. Once participants transitioned to the flat-road cycling phase, lasting 7–8 min, upbeat and cheerful music was introduced. This selection was intended to boost morale and assist participants in maintaining a steady pace during moderate-intensity cycling. For the uphill climbing phase, which spanned 8–9 min, the music shifted to a slower tempo but with energetic beats to motivate participants through the increased resistance and intensity of climbing (46). The goal was to provide a sense of perseverance while helping them maintain focus under physical strain. In contrast, during the 7–8 min of downhill virtual sprints, fast-paced, passionate rock music was chosen to energize participants and promote rapid cycling (47). Finally, the cool-down phase featured 7–8 min of soft, lyrical music to help lower participants’ heart rates and facilitate relaxation (48).

#### 2.2.4 Implementation plan

Both the experimental and control groups participated in cycling sessions three times per week for 45 min at a fixed college fitness venue. The control group adhered to a conventional indoor cycling routine without the use of VR goggles or music, while the experimental group engaged in a 12-week VR cycling program. Instructors provided standardized verbal commands throughout the sessions, ensuring consistent adjustments in cycling postures and frequencies as required by the exercise phases (e.g., warm-up, high-resistance climbs, sprints). The VR scenes were synchronized with specific exercise stages, with transitions guided by the instructors to ensure smooth alignment with the training plan. Table 1 provides a detailed outline of these VR scenes and exercise arrangements.

**Table 1:** Cycling exercise protocols for experimental and control groups.

Arrangement	Content	Duration	Speed per hour	Revolution per minute
Warm-up	Slow rhythm music, riding on a bike, holding one hand, the body of each joint muscles to warm up	6–7 min	20–30 km/h	80–100 rpm
Riding in a standing position on a flat road	1, 2, 3 alternate standing position riding, enter the VR scene flat road, adapt to riding, resistance increased to 3	7–8 min	35–45 km/h	100–120 rpm
High resistance climb	With three handlebars in hand, the VR scene enters the mountain road. The resistance of the virtual mountain road increases. When riding on the top of the mountain, the resistance slowly increases by 3–8.	8–9 min	15–25 km/h	60–80 rpm
Sitting sprint	Hold 1 position, VR scene enters the downhill section, virtual sprint, resistance drops to 0, feet parallel to the ground, legs swing at the fastest frequency.	7–8 min	50–70 km/h	130–150 rpm
Practice periodically	1, 2, and 3 positions alternate. The VR scene enters the road with potholes, resistance 3–6, intermittent riding, alternating sitting and standing positions.	9–10 min	30–40 km/h	90–110 rpm
Stretch	Enter the homing section with a one-position VR scene in hand, alternate sitting and standing positions, combine soothing music, and perform muscle stretching with 0–2 resistance.	7–8 min	15–25 km/h	70–90 rpm

Resistance points 0–10, 10 for the maximum resistance.

**Figure 3:**
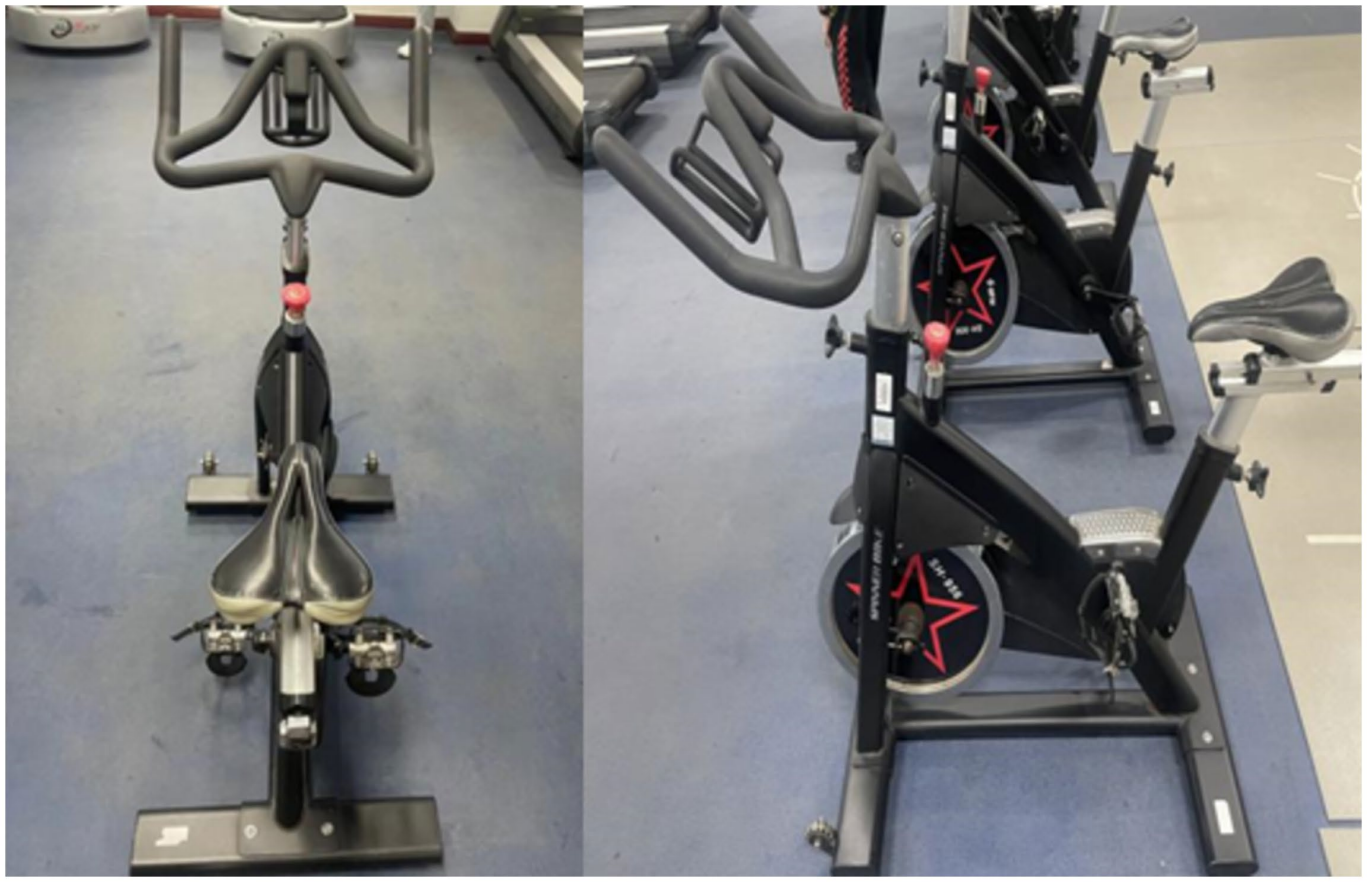
SHUA SH-956 dynamic bicycle used in the experiment.

Figure 3 shows the SHUA SH-956 (Made in China) dynamic bicycle used in the experiment. The dynamic bicycle is equipped with a resistance scale setting, located in the red knob area shown in the figure. This equipment was calibrated before each session to ensure resistance accuracy, with resistance adjustable across 10 levels. Participants adjusted resistance based on instructors’ commands during sessions, maintaining immersion by making adjustments without removing VR goggles. An additional experimenter monitored adjustments to minimize variation among participants and enhance the experiment’s reliability.

Throughout the study, key metrics—height, weight, waist and hip circumferences, BMI, waist-to-hip ratio, and lung capacity—were measured at the start of the study, at 6 weeks, and after 12 weeks. To maintain consistency in physical activity, participants in both groups were instructed to refrain from other aerobic or anaerobic exercises. Dietary monitoring was implemented via a dedicated WeChat group, where participants received feedback on their eating habits, along with simple nutritional recommendations to support balanced dietary intake. Additionally, participants were advised to minimize smoking and drinking to maximize training outcomes.

#### 2.2.5 Measurement instruments and protocols

The primary instruments utilized in this study included the InBody 770 body composition analyzer, a measuring tape, and a spirometer. The InBody 770 provided detailed assessments of participants’ height, weight, BMI, and visceral fat content, aiding in obesity analysis by integrating age-related data.

Measurements were taken at three intervals: before the experiment, at the 6-week mark, and at the 12-week conclusion. To ensure accuracy and consistency, all assessments were conducted at approximately 8 a.m., with participants in a fasting state. Trained personnel used the InBody 770 to record height, weight, and BMI, while participants, standing barefoot on the device, held the designated handles to complete the body composition scan. The device generated a comprehensive report including BMI and obesity metrics. Waist and hip circumference were measured manually using measuring tape, allowing for the calculation of the waist-to-hip ratio. Lung capacity was measured using a spirometer, with participants performing two breath tests, and the highest value was recorded.

### 2.3 Statistical analysis methods

All statistical analyses were performed using the statistical package Stata16.0. Initially, basic descriptive statistics for participants’ demographic and anthropometric characteristics, as well as each outcome variable, were calculated using means and standard deviations. Following this, an independent samples t-test was conducted to compare the mean differences of the primary outcome variables between the experimental and control groups across three distinct time periods. To evaluate the distributional differences in the outcome variables, the enhanced Kolmogorov–Smirnov test was utilized. Furthermore, the study employed a difference-in-differences model, controlling for both time and individual fixed effects, to rigorously assess the impact of the “Virtual Reality Cycling with Music” intervention on the target population.

#### 2.3.1 Kolmogorov–Smirnov test

When comparing different distributions, beyond assessing the differences in means, there is a growing emphasis on examining the entire distribution and its variations at different quantiles. The Kolmogorov–Smirnov (KS) test is a widely utilized method for assessing overall differences between two distributions. This non-parametric test, under the null hypothesis of equal cumulative distribution functions (CDFs), utilizes the maximum vertical distance between their empirical distribution functions as the test statistic. The hypothesis testing is performed through the asymptotic distribution of this statistic, which follows the Kolmogorov distribution. However, the KS test has two notable limitations: (1) It cannot adequately address differences at specific quantiles or values with a single hypothesis test; (2) Its sensitivity is uneven, being concentrated around the median of the distribution while showing reduced sensitivity in the tails, potentially leading to substantial inferential errors.

Goldman and Kaplan (49) introduced an enhanced version of the KS test to mitigate these issues. Their method, applicable under the assumptions that two samples are independently and identically distributed and mutually independent, and assuming a continuous (or partially discontinuous) overall distribution, offers several improvements over the traditional KS test: (1) By conducting multiple tests at different quantiles or values, this method allows for a more detailed comparison of distributional differences across various segments of the distributions. (2) The enhanced KS test maintains strong control over the family-wise error rate in multiple quantile testing. It ensures that the rate of multiple errors is kept below a predetermined level for all potential sample values. Achieving strong control implies that for any value where the two CDFs are equal, the multiple error rejection rate is less than the specified level. (3) The enhanced KS test addresses the issue of reduced sensitivity in the distribution tails, thus providing a more uniformly distributed statistical power across the entire distribution. These advancements ensure that the enhanced KS test offers a more comprehensive and sensitive approach to distribution comparison, particularly in capturing differences across the entire range of values.

#### 2.3.2 Difference-in-differences model

To rigorously assess impact of the “Virtual Reality Cycling with Music” intervention, it is essential to use the classic difference-in-differences (DID) model. This statistical approach isolates the causal effects of the intervention by examining the differential changes in outcomes over time between a treatment group and a control group. The DID model effectively controls for unobserved confounders and time-invariant factors that might otherwise bias the results, ensuring that the estimated effect of the intervention is both unbiased and consistent. The specific model specification is as follows:

$$y_{\mathrm{it}}=\alpha_{0}+\alpha_{1}\mathrm{grou}p_{i}+\alpha_{2}\mathrm{perio}d_{t}+\alpha_{3}\mathrm{grou}p_{i}*\mathrm{perio}d_{t}+\varepsilon_{\mathrm{it}}$$

In the model, $y_{\mathrm{it}}$ stands for the various outcome measures we are interested in observed for individual *i* at time period *t*, where *t* = 0 indicates the pre-experiment period and *t* = 1 indicates the post-experiment period. The outcome variables include weight, BMI, waist circumference, hip circumference, and vital capacity. To manage outliers and reduce variability within the data, logarithmic transformations were applied to these variables. This transformation also enables the analysis to be conducted in terms of semi-elasticity, facilitates more meaningful economic interpretations and insights. The variable $\mathrm{grou}p_{i}$ is a dummy variable for the experimental group, taking a value of 1 if the individual was subjected to the “Virtual Reality Cycling with Music” intervention and 0 otherwise. The variable $\mathrm{perio}d_{t}$ is a time dummy variable, with *t* = 0 denoting the pre-experiment period and *t* = 1 denoting the post-experiment period. Data from the mid-experiment phase were deliberately excluded to focus on the pre- and post-experiment periods. This approach enhances the reliability and interpretability of the results by reducing potential confounding factors that might have emerged during the mid-experiment phase. $\varepsilon_{\mathrm{it}}$ stands for the random error term. The variable $\mathrm{grou}p_{i}*\mathrm{perio}d_{t}$ denotes the interaction between a dummy variable for the experimental group and a time dummy variable. This interaction term captures the causal effect of the intervention on the experimental group, serving as the primary explanatory variable of interest in the model. The coefficient $\alpha_{3}$ denotes the causal effect of the intervention, which is the main focus of this study.

The DID model is based on a crucial fundamental assumption known as the parallel trends assumption, which posits that, in the absence of an intervention, the treatment and control groups would follow similar trends over time. To ensure this assumption holds true in our study, we implemented a randomized controlled trial. The rigorous randomization process ensures that any observed differences in outcomes can be attributed directly to the intervention, rather than pre-existing differences between the groups. We employ independent samples t-tests to test for baseline balance between the control and the experimental groups. As shown in Supplementary Table S1, the mean difference tests for all variables yielded *p*-values greater than 10%, indicating no significant baseline differences between the groups, thereby validating the success of the randomization process. This allows us to conclude that there is no evidence of a significant structural difference in overall respondent characteristics between the treatment and the control groups, ensuring the validity of comparisons between the two groups.

## 3 Results

### 3.1 Data and descriptive statistics

The research team conducted three observations of the key physical characteristic indicators of the subjects before, during, and after the experiment, obtaining a total of 234 valid observation values, of which 123 were from the experimental group and 111 were from the control group. The descriptive statistics of the main variables of the pre-experiment samples are shown in Table 2.

**Table 2:** Descriptive statistics of key physical characteristics pre-experiment.

Variable (abbrev.)	*N*	Mean	SD	Min	Max
Sex (1 = male)	78	0.58	0.50	0	1
Age	78	19.96	1.62	18	24
Height	78	1.69	0.09	1.48	1.93
Weight	78	82.62	8.38	65	102
Body Mass Index (bmi)	78	28.76	1.76	24.99	33.61
Waistline	78	96.87	5.00	88.7	114
Hipline	78	96.41	3.54	90.2	106.7
Waist-to-Hip Ratio (whr)	78	1.00	0.03	0.94	1.13
Forced Vital Capacity (fvc)	78	3,373.71	639.42	1,998	4,887

In this study, independent t-tests were employed to compare the mean values of various variables between the experimental and control groups at three distinct stages: pre-experiment (baseline characteristics), during the experiment, and post-experiment, as detailed in Supplementary Tables S1–S3. As illustrated in Supplementary Table S1, there were no significant differences in baseline characteristics, such as gender and age, between the experimental and control groups, ensuring the comparability of these groups at the study’s outset. Supplementary Table S2 illustrates significant variations in waist and hip circumferences recorded over the course of the experiment. Notably, the experimental group exhibited notable reductions, with waist circumference decreasing by 2.76 cm and hip circumference by 3.04 cm. These reductions were statistically significant, with *p*-values of less than 0.05 and 0.01, respectively. These findings suggest the preliminary efficacy of the intervention under investigation. Supplementary Table S3 elucidates notable post-experimental disparities across several variables, including weight, BMI, waist circumference, and hip circumference. Specifically, the experimental group exhibited a mean weight reduction of 3.15 kg, a finding that is statistically significant at the 10% level. Moreover, the experimental cohort demonstrated a decrease in BMI by 0.97, with statistical significance at the 5% level. Regarding waist circumference, the experimental group achieved an average reduction of 4.32 cm, which is statistically significant at the 1% level. Similarly, a reduction in hip circumference by 3.59 cm was observed in the experimental group, with this change also reaching statistical significance at the 1% level. These findings underscore the enduring impact of the intervention and its potential efficacy in modulating critical health indicators.

**Figure 4:**
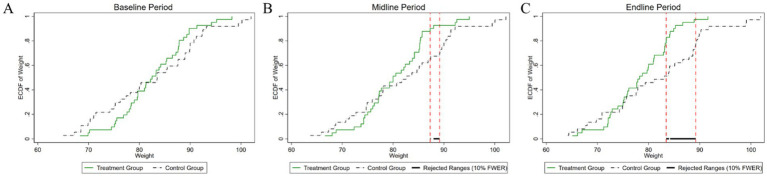
Changes in the distribution of weight across experimental stages: **(A)** baseline, **(B)** midline, **(C)** endline.

**Figure 5:**
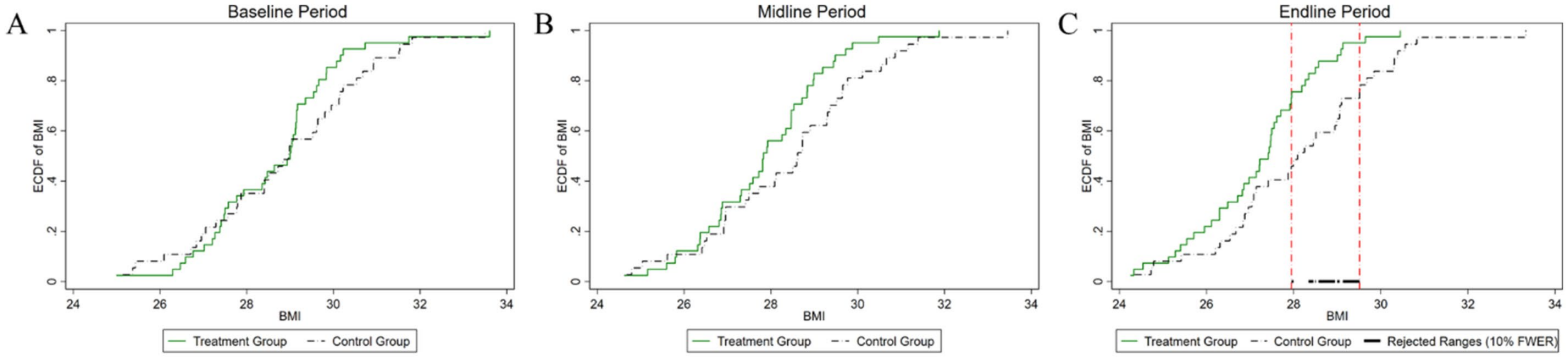
Changes in the distribution of BMI across experimental stages: **(A)** baseline, **(B)** midline, **(C)** endline.

**Figure 6:**
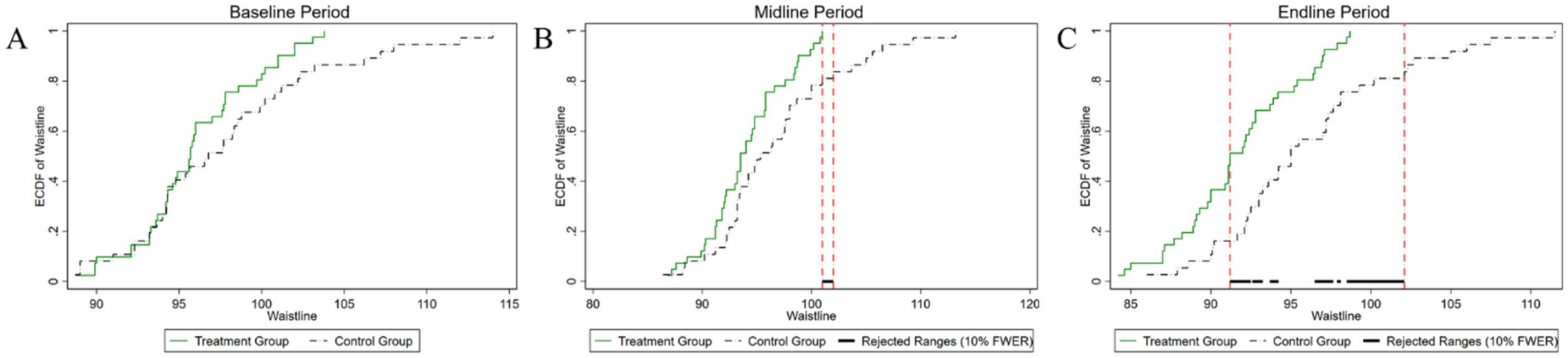
Changes in the distribution of waist circumference across experimental stages: **(A)** baseline, **(B)** midline, **(C)** endline.

**Figure 7:**
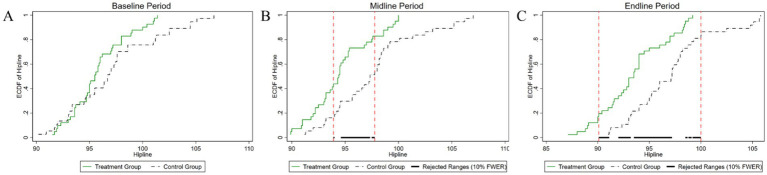
Changes in the distribution of hip circumference across experimental stages: **(A)** baseline, **(B)** midline, **(C)** endline.

**Figure 8:**
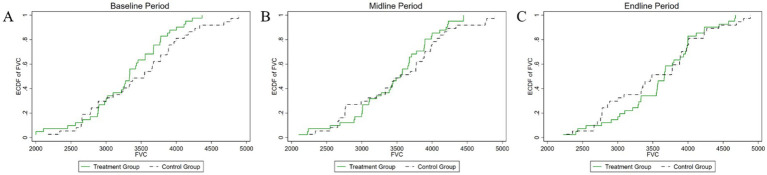
Changes in the distribution of vital capacity across experimental stages: **(A)** baseline, **(B)** midline, **(C)** endline.

Figures 4–8 were generated utilizing the enhanced version of the KS test. The figures are divided into three sections for clearer interpretation: (1) The far-left section, labeled as the ‘(A) baseline period’, represents the outcome variables of both the experimental and control groups before the experiment’s onset. This initial comparison establishes a reference point for subsequent changes. (2) The center section, referred to as the ‘(B) midline period’, illustrates the distributions of the outcome variables during the experiment. This period captures the dynamic changes and interactions within the groups under experimental conditions, offering insights into the immediate effects and the evolving trajectories of the variables. (3) The far-right section, known as the ‘(C) endline period’, displays the distributions of the outcome variables post-experiment, providing a comprehensive view of the final impact of the experimental intervention. By employing the enhanced KS test, we can rigorously assess the statistical significance of the differences observed across these periods, ensuring a robust interpretation of the experimental outcomes and the intervention’s effectiveness.

As illustrated in Figure 4, the baseline weight distributions for both the experimental and control groups exhibit a high degree of similarity. The *p*-value of the difference test of the weight distribution of the two groups is 0.418, which is well above the conventional 10% significance level. Consequently, we fail to reject the null hypothesis, indicating that there is no statistically significant difference in the weight distributions between the experimental and control groups at the initial stage of the study. During the mid-experiment period, a statistically significant difference in weight distribution between the experimental and control groups was observed within a specific weight range. This range, delineated by two vertical lines, spans from 87 kg to 89 kg. The *p*-value for this hypothesis test was computed to be 0.072, which indicates statistical significance at the 10% level. This particular range highlights where the most pronounced discrepancies in weight distributions are observed, shedding light on the critical impact of the experimental intervention on the subjects’ weights. These findings suggest that the intervention may be particularly effective within this narrow weight range, providing valuable insights into its efficacy and potential mechanisms of intervention. In the post-experiment period, the weight variable continued to show a statistically significant difference in distribution between the two groups. The interval defined by the two vertical lines, spanning from 83 kg to 89 kg, marks the region where these differences are most pronounced. The *p*-value obtained from the statistical test for the difference in weight distribution is 0.01, indicating statistical significance at the 1% level. Compared to the mid-experiment period, this interval has expanded in the post-experiment period, highlighting an intensification of the experimental effect. This expansion suggests that the intervention’s impact has become more pronounced over time, underscoring the growing influence of the intervention on weight distribution.

As depicted in Figure 5, the pre-experiment period reveals a *p*-value of 0.519 for the difference test of BMI distribution between the two groups. This value exceeds the conventional 10% significance level, indicating no statistically significant difference between the groups at baseline. During the mid-experiment period, the *p*-value was 0.227, again surpassing the 10% significance level. Consequently, this result does not provide evidence of a significant difference in BMI distribution during this period. However, in the post-experiment period, the *p*-value decreased to 0.064, which falls below the 10% significance level. This suggests a statistically significant difference in BMI distribution between the two groups, with the significant interval primarily concentrated in the range of 27–29. These findings indicate that while the experimental intervention did not yield immediate significant changes in BMI distribution during the early and mid-stages, its effects became more pronounced and statistically significant in the post-experiment period.

As illustrated in Figure 6, the initial comparative analysis of waist circumference distributions between the two groups yielded a *p*-value of 0.268. This *p*-value exceeds the 10% significance level, signifying that there was no statistically significant difference in waist circumference between the groups at the outset of the experiment. During the mid-experiment period, the *p*-value was determined to be 0.056, indicating statistical significance at the 10% significance level. This result suggests a notable difference in the distributions of waist circumference, particularly within the range of 101–102 cm. Following the conclusion of the experiment, the *p*-value further decreased to 0.009, signifying a statistically significant difference at the 1% significance level. The most notable differences in waist circumference distribution were observed within the range of 90–102 cm. Upon completion of the experiment, the *p*-value further decreased to 0.009, indicating a statistically significant difference at the 1% significance level. The most pronounced variations in waist circumference distribution were observed in the 90–102 cm interval. This result underscores the increasing impact of the experimental intervention on waist circumference over time.

As shown in Figure 7, the temporal evolution of hip circumference distributions between the experimental and control groups is comprehensively depicted. Initially, before the experiment, the *p*-value for testing the difference in hip circumference distributions between the two groups was 0.134, exceeding the conventional 10% significance level. Thus, we did not reject the null hypothesis, indicating no statistically significant difference in hip circumference between the groups at baseline. During the mid-experiment period, the *p*-value for the test of distributional differences decreased markedly to 0.002, reaching statistical significance at the 1% level. This significant divergence was predominantly observed within the hip circumference range of 93–98 cm. In the post-experimental period, the *p*-value remained at 0.002, maintaining significance at the 1% level. The interval exhibiting statistically significant differences expanded to encompass the range from 90 cm to 100 cm. This expansion indicates an intensification and broadening of the intervention’s impact over time, suggesting an increasing efficacy and a more pervasive influence of the intervention on hip circumference distribution as the experiment progressed.

As shown in Figure 8, the initial analysis found a *p*-value of 0.695 for the test of differences in vital capacity between the experimental and control groups. This indicates no statistically significant difference between the groups at the start of the experiment. During the mid-experiment period, the *p*-value remained high at 0.71, well above the 10% significance level, which means the null hypothesis cannot be rejected, and no significant difference in vital capacity distribution was observed. In the post-experiment period, the *p*-value was 0.519, still above the 10% significance level, indicating no statistically significant difference in vital capacity distribution between the groups after the intervention. To better understand the intervention’s impact on vital capacity, it is essential to apply an empirical model for a more rigorous examination of its effects.

### 3.2 Empirical estimation

To effectively address unobservable individual heterogeneity, we employed a fixed-effects estimation method within panel data models to accurately estimate the DID model. As presented in Table 3, the findings indicate that VR cycling has a significant impact on fat reduction, demonstrating marked improvements across various body metrics. Specifically, the intervention results in a 4.1% reduction in weight, a 2.8% decrease in BMI, a 2.8% decrease in waist circumference, and a 2.5% decrease in hip circumference. Moreover, there was a reduction of 0.016 units in the waist-to-hip ratio. Since this ratio is not logarithmically transformed, there is no need to convert it to percentage terms. Additionally, vital capacity shows a significant enhancement, with a 7.2% increase attributed to the VR cycling intervention with music. These findings underscore the potential of this innovative intervention as a valuable tool in combating obesity and promoting overall health.

**Table 3:** Model estimation results for physical metrics in VR cycling intervention.

	(1)	(2)	(3)	(4)	(5)	(6)
	ln_weight	ln_bmi	ln_waistline	ln_hipline	whr	ln_fvc
Group*Period	−0.041^***^	−0.028^***^	−0.028^***^	−0.025^***^	−0.016^***^	0.072^***^
	(0.004)	(0.004)	(0.003)	(0.003)	(0.003)	(0.010)
Period = 1	−0.014^***^	−0.026^***^	−0.018^***^	−0.000	−0.004^**^	0.017^*^
	(0.002)	(0.002)	(0.002)	(0.002)	(0.002)	(0.009)
Constant	4.409^***^	3.357^***^	4.572^***^	4.568^***^	0.998^***^	8.105^***^
	(0.001)	(0.001)	(0.001)	(0.001)	(0.001)	(0.003)
Observations	156	156	156	156	156	156
Adjusted *R*^2^	0.844	0.855	0.867	0.645	0.638	0.687

*, **, and *** indicate significance at the 10, 5, and 1% level, respectively. Standard errors in parentheses.

To further confirm the robustness of our empirical findings, we performed regression analyses stratified by gender, as detailed in Supplementary Tables S4. The results consistently demonstrated that the significance levels and directional effects of the key explanatory variables were similar across both male and female subgroups. This uniformity underscores the reliability and validity of our estimated coefficients, suggesting that the observed relationships are not influenced by gender-specific dynamics but are broadly applicable across different gender groups.

In contrast, the “Virtual Reality Cycling with Music” intervention had a more significant impact on males compared to females. Specifically, males showed significant reductions in body weight, BMI, and waist-to-hip ratio, whereas females experienced more notable reductions in waist and hip circumferences. These differences can be attributed to inherent physiological differences between genders. Typically, obese males tend to have an “apple-shaped” body, characterized by a larger abdomen and hips with most fat concentrated in the abdominal area, resulting in a waist-to-hip ratio greater than 1. On the other hand, despite some female participants being obese before the experiment, their unique physiological curves often lead to a lower waist-to-hip ratio, with some females displaying a “pear-shaped” body (50).

### 3.3 Further analysis

As illustrated in Supplementary Table S5, the average weight of males in the experimental group slightly exceeds that of females, while the average BMI of females is marginally higher, consistent with general trends. Females also exhibit slightly smaller average waist circumference, hip circumference, and waist-to-hip ratio, which can be attributed to physiological differences between genders. Notably, males tend to have a waist-to-hip ratio exceeding 1. This ratio is a crucial indicator of central obesity (51), which is associated with increased risks of cardiovascular disease and diabetes, particularly among severely obese individuals (52). Therefore, participants in the experimental group should focus on weight control and overall health improvement through a balanced diet and regular exercise.

Supplementary Table S6 details the results of a 12-week intervention, revealing significant reductions in various health indicators for the experimental group. Specifically, participants experienced substantial decreases in weight (5.25%), BMI (5.22%), waist circumference (4.44%), hip circumference (2.44%), and waist-to-hip ratio (2.1%) (all *p* < 0.01). Moreover, there was a significant increase in vital capacity by 9.15% (*p* < 0.05). These improvements are attributed to the intervention that included prolonged periods of standing and cycling in a virtual reality environment. This type of exercise engages multiple parts of the body, particularly the core areas such as the waist and hips. The sessions consisted of 45–50 min of indoor cycling in a controlled environment, which promotes calorie expenditure comparable to jogging. The vivid and engaging VR cycling sessions, combined with the rhythmic pedaling on the stationary bike, enabled the experimental group to achieve significant weight loss. Furthermore, after 12 weeks of VR cycling exercise, there was a noticeable increase in vital capacity. This whole-body exercise, enhanced by rhythmic music and the immersive VR experience, significantly improved cardiovascular function. As a result, the experimental group exhibited substantial improvements in key health indicators after the 12-week VR cycling regimen (53).

Supplementary Tables S7, S8 provide detailed evidence of the intervention’s effectiveness in improving key health indicators for both female and male participants. These tables illustrate significant improvements in various metrics, demonstrating the positive impact of the intervention on the health outcomes of the study population. Specifically, Supplementary Table S7 highlights significant health improvements in female participants. Notably, weight decreased by approximately 4.87% (*p* < 0.05), BMI by 4.91% (*p* < 0.05), waist circumference by 5.26% (*p* < 0.01), and hip circumference by 3.80% (*p* < 0.01). Furthermore, the waist-to-hip ratio reduced by 1.51% (*p* < 0.05), and vital capacity increased by 9.82% (*p* < 0.1). Similarly, Supplementary Table S8 highlights the health improvements observed in male participants. Weight decreased by 5.46% (*p* < 0.01), BMI by 5.42% (*p* < 0.01), waist circumference by 3.93% (*p* < 0.01), and hip circumference by 1.59% (*p* < 0.05). Additionally, the waist-to-hip ratio reduced by 2.39% (*p* < 0.01), and vital capacity increased by 8.82% (*p* < 0.01).

It is noteworthy that females generally have a higher innate fat content than males before testing. Throughout the experiment, VR cycling was found to more effectively target core areas such as the waist, hips, and thighs. Females showed a greater percentage decrease in waist and hip circumference, while males expended more abdominal fat, resulting in a more significant improvement in the waist-to-hip ratio compared to females. Additionally, females typically have lower vital capacity before the experiment, and after 12 weeks of cycling exercise, the increase in vital capacity in females is more significant. On the other hand, males have higher vital capacity before the experiment, with some obese males having vital capacity higher than the standard value before the experiment. Therefore, the increase in vital capacity in males is not as significant compared to females. Supplementary Figure S2 provides a more intuitive graphical explanation.

Tables 4, 5 present participants’ feedback on the two cycling training programs for the experimental and control groups, respectively. In the control group, which followed the traditional cycling program, only a small proportion of participants—approximately 20%—reported positive experiences across enjoyment, willingness to continue, and overall satisfaction (6, 7, and 7 participants, respectively). A slightly larger subset, around 35%, indicated moderate levels of experiences (11, 13, and 14 participants). However, the majority—50% of participants—expressed a poor experience, indicating a generally negative perception of traditional cycling. Regarding willingness to continue participating, 17 participants expressed a relatively poor experience, directly affecting their intention to engage in future sessions. Similarly, acceptance and satisfaction levels were notably low, with 16 participants indicating dissatisfaction, potentially linked to environmental and infrastructural factors.

**Table 4:** Participants’ feedback on the VR cycling program with music in the experimental group.

	Imagery	Consistency	Immersion	Acceptance and satisfaction of VR cycling
Relatively good	24	40	26	29
Generally good	13	8	13	9
Relatively poor	4	3	2	4

**Table 5:** Participants’ feedback on the traditional cycling program in the control group.

	Interestingness	Whether to continue later	Acceptance and satisfaction with traditional riding
Relatively good	6	7	7
Generally good	11	13	14
Relatively poor	20	17	16

In contrast, the VR cycling intervention with music produced markedly different outcomes. Table 4 shows that 29 participants in the experimental group reported high levels of acceptance and satisfaction, attributing their positive experiences to the immersive environment created by VR technology. The interactive features of the VR system, such as adjustable cycling routes, speed, direction, and gradient, engaged participants in a highly immersive three-dimensional environment. This level of engagement suggests that VR cycling offers a more dynamic and appealing exercise experience compared to traditional cycling methods (54). Moreover, only four participants rated their VR experience as moderate, citing limitations in visual quality and immersion when wearing the VR goggles. These insights underscore the potential for future technological advancements to further improve the effectiveness and appeal of VR-based exercise.

In summary, traditional cycling received limited positive feedback, whereas VR cycling demonstrated substantially higher engagement and satisfaction. This contrast highlights the potential of VR-based exercise programs to encourage sustained physical activity, particularly among individuals less inclined toward conventional exercise methods.

## 4 Discussion

This study comprehensively examined the physiological impacts of integrating VR cycling with music on obesity management among college students, aiming to develop innovative and effective interventions for obesity management within this demographic (55). The findings demonstrate that while both the experimental and control groups experienced fat loss, participants in the experimental group exhibited more significant reductions in weight, BMI, waist and hip circumference, and waist-to-hip ratio. These enhanced outcomes can be attributed to the increased motivation and engagement fostered by the combination of VR and music, which promoted higher levels of physical activity and greater involvement of multiple body regions in fat-burning processes, resulting in more pronounced fat loss (32–34). Although the exercise intensity remained consistent between the two groups, participants in the control group reported a more monotonous training experience, leading to boredom and passivity. As a result, while some fat loss was achieved in the control group, it was not as substantial as that in the experimental group. Furthermore, the experimental group showed a notable improvement in vital capacity, underscoring the broader health benefits of this intervention.

Psychologically, the synergistic effect of music and VR created a more enjoyable and sustainable exercise experience (56). Music, through rhythmic entrainment, helps synchronize the participants’ movements with the tempo, optimizing motor coordination and improving energy efficiency (32–34). Additionally, music promotes attention dissociation, diverting focus away from sensations of fatigue and discomfort, and toward external auditory stimuli. This mechanism helps lower perceived exertion, enabling participants to exercise for longer periods with greater endurance and improved emotional states (32–35). Complementing these benefits, the immersive qualities of VR further amplify the psychological advantages by offering engaging visual stimuli, often simulating outdoor environments. These virtual landscapes not only mitigates the monotony associated with traditional indoor exercise but also enhances cognitive focus and enjoyment, creating a more dynamic and appealing exercise routine (57). These combined effects—heightened enjoyment, reduced perceived effort, and sustained motivation—are particularly effective in addressing common barriers to regular physical activity among college students, such as lack of interest and engagement.

Scholars have made significant strides in integrating visual stimuli into exercise programs to enhance the psychological and physiological outcomes of physical activity. For example, research found that Jones et al. used footage of a country park in conjunction with an indoor cycling ergometer (34). They reported that, compared to video and control conditions, music and music video conditions elicited more positive exercise-related effects. Barreto-Silva et al. extended this line of inquiry by incorporating pleasant, unpleasant, and neutral road scenes during cycling tasks (58). Participants exposed to pleasant imagery—such as cyclists navigating downhill routes—demonstrated a notable 5% increase in cycling speed, alongside a significant reduction in ratings of perceived exertion when compared to unpleasant scenes. These studies collectively underscore the potential for tailored visual environments to modulate not only performance metrics but also subjective exercise experiences, which could have broad implications for the design of future exercise programs aimed at improving adherence and enjoyment.

From a physiological perspective, VR cycling with music offers more pronounced fat-reduction effects compared to traditional cycling. Studies indicate that regular and rhythmic physical exercise not only reduces fat tissue but also enhances overall metabolism (59). This enhanced physiological effect can be attributed to several interrelated factors. Firstly, the immersive qualities of VR and the motivational influence of music combine to create a multisensory experience that enhances physical engagement. This synergy stimulates a wider range of body movements, increasing overall energy expenditure (32–34). Secondly, the positive emotional states induced by VR and music potentially modulate neuroendocrine responses (60), such as the release of endorphins and other happy hormones (61), which further facilitated fat metabolism.

In terms of public health implications, this study highlights the importance of integrating psychological and behavioral factors into obesity management strategies (62, 63). By incorporating elements such as music and VR, the study presents a novel approach that can make physical activity more appealing, addressing common barriers to exercise in college students. VR cycling offers a distinct advantage over traditional cycling exercise by creating an immersive and dynamic environment that not only mitigates monotony but also adapts the difficulty and duration of exercise sessions to optimize fat loss outcomes. This innovative approach has the potential to revolutionize exercise interventions by making them more enjoyable and effective, thereby improving adherence and enhancing overall health outcomes (51). As such, this method could become a valuable addition to public health interventions aimed at combating obesity, especially in younger populations who may be more receptive to technologically enhanced and engaging exercise formats.

In conclusion, the discussion from three perspectives and comparisons with previous studies demonstrate that VR cycling combined with music is an innovative approach that has been relatively underexplored in earlier research. This combination creates a fully immersive experience for participants using VR technology. By wearing VR glasses, participants are transported into a three-dimensional virtual environment, enhancing the overall experience. This technology holds the potential for continuous improvement and evolution, providing opportunities for further refinement and innovation in the fitness field.

## 5 Conclusion

This study rigorously evaluated the impact of VR cycling combined with music on simple obesity in college students through a well-designed randomized controlled trial. The trial recruited college students diagnosed with simple obesity, randomly assigning them to either an experimental group (VR cycling with music) or a control group (traditional cycling). Over a 12-week period, participants engaged in thrice-weekly cycling sessions, with key physiological indicators such as weight, BMI, waist circumference, and vital capacity closely monitored. The experimental data were analyzed using independent samples t-test, KS test and a DID model. The main regression results demonstrated that VR cycling combined with music significantly enhanced fat reduction and body shaping compared to traditional methods. Specifically, the experimental group showed marked improvements in weight, BMI, waist and hip circumference, with reductions ranging from 2.5 to 4.2%, and an increase in vital capacity by 7.2%. Importantly, gender-stratified regression analyses confirmed the robustness of these findings across male and female subgroups. While the intervention produced significant reductions in body weight, BMI, and waist-to-hip ratio in males, females experienced more notable decreases in waist and hip circumferences, reflecting inherent physiological differences between genders. These findings highlight the efficacy of VR cycling with music as a novel and engaging method for obesity management. Furthermore, the psychological benefits of the intervention were clear, with participants reporting enhanced exercise enjoyment and persistence. This outcome is likely due to the immersive and dissociative effects of the VR and music combination, which mitigates the monotony often associated with traditional exercise routines.

## 6 Limitations and future directions

While this study offers significant insights, it has certain limitations that should be acknowledged. First, the sample size is relatively small and focuses solely on college students from a specific region in China, which may restrict the generalizability of the results. To enhance the external validity, future research should consider incorporating a larger and more diverse sample that spans various regions and demographic groups.

Second, the study only focuses primarily on short-term effects, leaving the long-term impact of VR cycling combined with music on obesity management unexplored. To fully understand the enduring benefits and potential long-term effects, longitudinal studies are essential. Moreover, this study did not account for variations in psychological and physiological responses to different types of music and VR environments, which could be crucial for optimizing intervention strategies. Future research should explore these aspects to tailor interventions more effectively based on individual preferences and responses.

Finally, the use of VR cycling technology in this study presents certain limitations in terms of cost, accessibility, maintenance, and sustainability. The VR headset used in the experiment was of moderate quality, which may have affected participants’ sense of immersion, realism, and interactivity. Although VR technology can offer engaging and immersive experiences, the cost of purchasing high-quality VR equipment (head-mounted displays, software, etc.) may limit its widespread adoption, particularly in budget-constrained public health programs. Additionally, in low-income or resource-limited regions, VR technology may not be accessible to all populations, and the demand for trained personnel may not be adequately met. The ease of access to and use of VR devices by the target audience, the regular maintenance of VR systems, and challenges such as software updates, hardware repairs, and the replacement of consumables could pose additional difficulties. Therefore, whether the long-term use of VR as a public health intervention is economically and technically sustainable requires further research and exploration.

## Data Availability

The raw data supporting the conclusions of this article will be made available by the authors, without undue reservation.
