# Supplementary material for: The effect of Virtual Reality Cycling with Music on simple obesity in college students: evidence from a randomized controlled trial in China
Source: Front Public Health. 2024 Nov 21;12:1466142. doi: 10.3389/fpubh.2024.1466142 (PMC11617581; doi:10.3389/fpubh.2024.1466142)

Supplementary Material

**Tables:**

**Table S1 Comparison of Mean Values of Variables between the Experimental and Control Groups (Before the Experiment)**

| Variables | Control group | Mean1 | Treatment group | Mean2 | Mean Difference |
| --- | --- | --- | --- | --- | --- |
| sex | 37 | 0.54 | 41 | 0.61 | -0.07 |
| age | 37 | 19.78 | 41 | 20.12 | -0.34 |
| height | 37 | 1.69 | 41 | 1.70 | -0.01 |
| weight | 37 | 82.54 | 41 | 82.70 | -0.16 |
| bmi | 37 | 28.87 | 41 | 28.65 | 0.22 |
| waistline | 37 | 97.82 | 41 | 96.02 | 1.80 |
| hipline | 37 | 97.07 | 41 | 95.80 | 1.27 |
| whr | 37 | 0.99 | 41 | 1.00 | -0.01 |
| fvc | 37 | 3464.14 | 41 | 3292.10 | 172.04 |

Note：*, **, and *** indicate significance at the 10%, 5%, and 1% level respectively.

**Table S2 Comparison of Mean Values of Variables between the Experimental and Control Groups (During the Experiment)**

| **Variables** | **Control group** | **Mean1** | **Treatment group** | **Mean2** | **Mean Difference** |
| --- | --- | --- | --- | --- | --- |
| **weight** | 37 | 82.01 | 41 | 80.37 | 1.65 |
| **bmi** | 37 | 28.42 | 41 | 27.85 | 0.58 |
| **waistline** | 37 | 96.77 | 41 | 94.01 | 2.76** |
| **hipline** | 37 | 97.63 | 41 | 94.60 | 3.04*** |
| **whr** | 37 | 0.99 | 41 | 0.99 | -0.00 |
| **fvc** | 37 | 3513.11 | 41 | 3453.63 | 60.47 |

Note：*, **, and *** indicate significance at the 10%, 5%, and 1% level respectively.

**Table S3 Comparison of Mean Values of Variables between the Experimental and Control Groups (After the Experiment)**

| **Variables** | **Control group** | **Mean1** | **Treatment group** | **Mean2** | **Mean Difference** |
| --- | --- | --- | --- | --- | --- |
| **weight** | 37 | 81.41 | 41 | 78.26 | 3.15* |
| **bmi** | 37 | 28.13 | 41 | 27.16 | 0.97** |
| **waistline** | 37 | 96.07 | 41 | 91.75 | 4.32*** |
| **hipline** | 37 | 97.06 | 41 | 93.47 | 3.59*** |
| **whr** | 37 | 0.99 | 41 | 0.98 | 0.010 |
| **fvc** | 37 | 3523.22 | 41 | 3593.17 | -69.95 |

Note：*, **, and *** indicate significance at the 10%, 5%, and 1% level respectively.

**Table S4 Regression Results for Gender Groups**

|  | **LN_Weight** | | **LN_BMI** | | **LN_Waistline** | | **LN_Hipline** | | **WHR** | | **LN_FVC** | |
| --- | --- | --- | --- | --- | --- | --- | --- | --- | --- | --- | --- | --- |
|  | **Male** | **Female** | **Male** | **Female** | **Male** | **Female** | **Male** | **Female** | **Male** | **Female** | **Male** | **Female** |
| **Group*Period** | -0.043*** | -0.037*** | -0.032*** | -0.022*** | -0.023*** | -0.035*** | -0.018*** | -0.036*** | -0.018*** | -0.012** | 0.075*** | 0.070*** |
|  | (0.005) | (0.006) | (0.005) | (0.005) | (0.004) | (0.005) | (0.003) | (0.004) | (0.003) | (0.004) | (0.017) | (0.010) |
| **Period=1** | -0.014*** | -0.014** | -0.024*** | -0.028*** | -0.017*** | -0.019*** | 0.002 | -0.003 | -0.006** | -0.003 | 0.010 | 0.025*** |
|  | (0.003) | (0.004) | (0.003) | (0.004) | (0.002) | (0.003) | (0.002) | (0.003) | (0.002) | (0.003) | (0.016) | (0.005) |
| **Constant** | 4.471*** | 4.324*** | 3.350*** | 3.367*** | 4.583*** | 4.557*** | 4.572*** | 4.563*** | 1.006*** | 0.987*** | 8.235*** | 7.928*** |
|  | (0.001) | (0.001) | (0.001) | (0.001) | (0.001) | (0.001) | (0.001) | (0.001) | (0.001) | (0.001) | (0.004) | (0.003) |
| **Observations** | 90 | 66 | 90 | 66 | 90 | 66 | 90 | 66 | 90 | 66 | 90 | 66 |
| **Adjusted R2** | 0.844 | 0.843 | 0.849 | 0.870 | 0.861 | 0.904 | 0.625 | 0.833 | 0.723 | 0.500 | 0.593 | 0.852 |

Notes：*, **, and *** indicate significance at the 10%, 5%, and 1% level respectively. Standard errors in parentheses.

**Table S5 Descriptive statistics**

| **Var name** | **Total** | | **Female** | | **Male** | |
| --- | --- | --- | --- | --- | --- | --- |
|  | **Mean** | **SD** | **Mean** | **SD** | **Mean** | **SD** |
| **age** | 20.122 | 1.833 | 20.000 | 1.506 | 20.200 | 2.041 |
| **height** | 1.699 | 0.079 | 1.626 | 0.057 | 1.746 | 0.051 |
| **pre_weight** | 82.699 | 6.830 | 77.053 | 4.861 | 86.312 | 5.305 |
| **pre_bmi** | 28.652 | 1.580 | 29.165 | 1.813 | 28.324 | 1.348 |
| **pre_waistline** | 96.015 | 3.694 | 93.912 | 2.955 | 97.361 | 3.528 |
| **pre_hipline** | 95.802 | 2.668 | 94.350 | 1.763 | 96.732 | 2.759 |
| **pre_whr** | 1.002 | 0.014 | 0.995 | 0.017 | 1.006 | 0.010 |
| **pre_fvc** | 3292.098 | 582.268 | 2758.500 | 435.236 | 3633.600 | 367.919 |
| **N** | 41 | | 16 | | 25 | |

PS: N represents the number of observed values, Mean represents the mean value, SD represents the standard deviation.

**Table S6 Differences in indicators before and after the experiment**

| **Var name** | **mean(pre_experiment)** | **mean(post_experiment)** | **mean-diff** | **t** |
| --- | --- | --- | --- | --- |
| **weight** | 82.699 | 78.361 | 4.338*** | 3.040 |
| **BMI** | 28.652 | 27.157 | 1.495*** | 4.491 |
| **waistline** | 96.015 | 91.751 | 4.264*** | 5.118 |
| **hipline** | 95.802 | 93.466 | 2.337*** | 3.571 |
| **whr** | 1.002 | 0.981 | 0.020*** | 6.376 |
| **fvc** | 3292.098 | 3593.171 | -301.073** | -2.297 |

Note: *, ** and *** represent the significance level of 10%, 5% and 1% respectively.

**Table S7 Differences in indicators before and after female experiments**

| **Var name** | **mean(pre_experiment)** | **mean(post_experiment)** | **mean-diff** | **t** |
| --- | --- | --- | --- | --- |
| **weight** | 77.053 | 73.303 | 3.750** | 2.260 |
| **BMI** | 29.165 | 27.734 | 1.431** | 2.473 |
| **waistline** | 93.912 | 88.969 | 4.944*** | 4.769 |
| **hipline** | 94.350 | 90.769 | 3.581*** | 5.263 |
| **whr** | 0.995 | 0.980 | 0.015** | 2.658 |
| **fvc** | 2758.500 | 3029.500 | -271.000* | -1.724 |

Note: *, ** and *** represent the significance level of 10%, 5% and 1% respectively.

**Table S8 Differences in indicators before and after male experiments**

| **Var name** | **mean(pre_experiment)** | **mean(post_experiment)** | **mean-diff** | **t** |
| --- | --- | --- | --- | --- |
| **weight** | 86.312 | 81.598 | 4.715*** | 3.373 |
| **BMI** | 28.324 | 26.788 | 1.537*** | 4.062 |
| **waistline** | 97.361 | 93.532 | 3.829*** | 3.959 |
| **hipline** | 96.732 | 95.192 | 1.540** | 2.031 |
| **whr** | 1.006 | 0.982 | 0.024*** | 6.605 |
| **fvc** | 3633.600 | 3953.920 | -320.320*** | -3.095 |

Note: *, ** and *** represent the significance level of 10%, 5% and 1% respectively.

**Figures:**

**Figure S1 Highway scene (departure section, forest section, night section)**


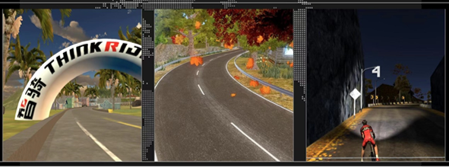


**Figure S2 Differences in indicators before and after the experiment (by sex)**


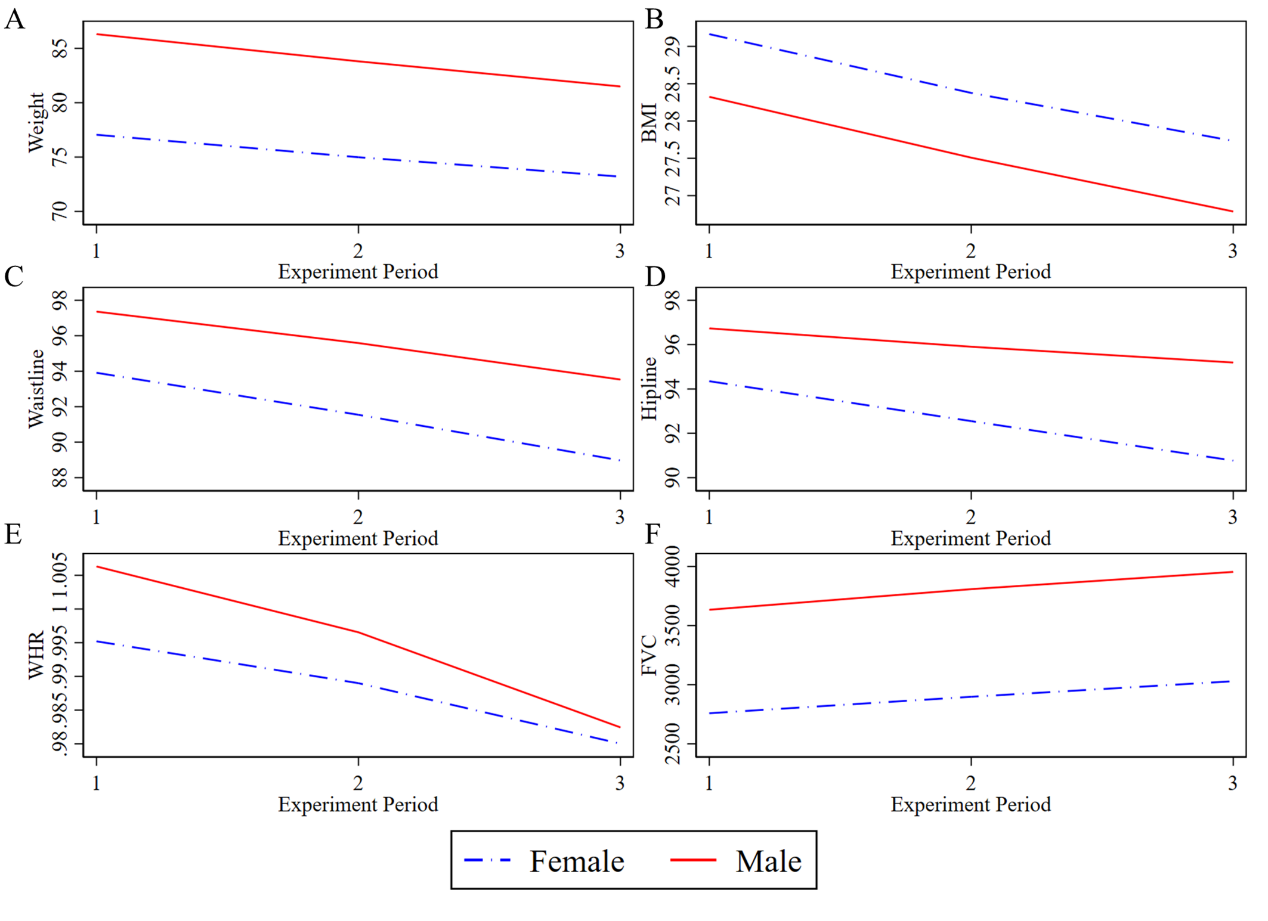

Supplement: Supplementary file 1 [file Data_Sheet_1.docx]
